# A novel machine learning approach to analysis of electroosmotic effects and heat transfer on Multi-phase wavy flow

**DOI:** 10.1038/s41598-025-18124-5

**Published:** 2025-10-22

**Authors:** Muhammad Naeem Aslam, Arshad Riaz, Muhammad Sarmad Arshad, A. AlZubaidi, Rida Maqsood, Abdullah M. Al-nahari, Mohamed Kallel

**Affiliations:** 1 Department of Mathematics, Lahore Garrison University, Lahore, 54770 Pakistan; 2 Department of Mathematics, Division of Science and Technology, University of Education, Lahore, 54770 Pakistan; 3 Department of Mathematics, College of Science, King Khalid University, 61413 Abha, Saudi Arabia; 4 School of Mathematics, Minhaj University, Lahore, 54770 Pakistan; 5 Department of Financial and Banking, Hodeidah University, Al Hudaydah, Yemen; 6 Department of Physics, College of Science, Northern Border University, Arar, Saudi Arabia

**Keywords:** Heat transfer, Wavy flow, Morlet neural networks, Particle swarm, Tanh activation function, Applied mathematics, Fluid dynamics

## Abstract

In this contribution, a novel hybrid approach involving artificial neural networks (ANNs) and heuristic algorithms is employed for Hall currents and electromagnetic effects analysis for a multi-phase wavy flow. The governing partial differential equations (PDEs) for flow dynamics are reduced into a corresponding system of ordinary differential equations (ODEs) with a pertinent transformation technique. The novelty of this work is combination of Morlet wavelet and hyperbolic tangent (Tanh) functions is employed as an activation function in artificial neural networks (ANNs) to effectively capture nonlinear behavior for flow dynamics. The novel effective Morlet wavele Tanh neural networks (MTNNs) based fitness function is formulated for solution estimation of the model. The weights and biases of MTNNs are optimized with a global searching technique by particle swarm optimization (PSO). Numerical solution of ODEs is also obtained through Python physics informed neural networks (PINNs) with Adam optimizer for validation of the proposed solutions. Statistical analysis involving histogram visualizations, probability plots, and boxplots is performed for accuracy, robustness, convergence, and stability evaluation of the proposed solution with respect to crucial error measures such as cost function, absolute error, and mean squared error (MSE). The MSE values for velocity and temperature range from $\:{10}^{-07}$ to $\:{10}^{-09}$ and $\:{10}^{-06}$ to $\:{10}^{-09}$, respectively. Graphical analysis reveals that flow velocity and thermal distributions are influenced directly by electroosmotic factor but are affected inversely with the applied magnetic field. The proposed MTNNs yield results that closely align with those obtained using PINNs.

## Introduction

Electroosmotic effects and heat transfer on multi-phase wavy flow play a significant role in different era in chemical, mechanical, nuclear and biological systems. Multi-phase flows involve the simultaneous movement of different fluid phases, such as gas, liquid, or solid particles, which can exhibit complex interactions in wavy. Heat transfer in such systems is crucial for maintaining thermal stability, electroosmosis, the movement of fluids under the influence of an electric field, can influence the flow behavior and microfluidic applications. The multi-phase wavy flow modeled in the form of nonlinear partial/ordinary differential equations (PDEs/ODEs). The term nanofluid originally proposed by Choi et al.^1^ to describe a fluid in which nanoscale particles are suspended in a base fluid with low thermal conductivity, such as oil, ethylene glycol, or water. Goswami et al.^2^ examined a mathematical model to study how two different types of fluids move via a microchannel with respect to electro-osmotic forces. In that mathematical model, the fluid at the core behaves according to a power-law model, which means that its flow depends non-linearly on the applied force. Jaffrin et al.^3^ presented a review article on peristaltic pumping, focusing on several important aspects, such as different flow geometries, the ratio of the wave amplitude to the channel height (amplitude ratio), the wavelength of the waves driving the flow, and the Reynolds number, which indicates whether the flow is laminar or turbulent. Kumar et al.^4^ studied how radiative heat transfer affects the flow of a nanofluid in the presence of a magnetic field along a vertical surface. Magneto-hydrodynamics (MHD) tackle with the behavior of electrically conducting fluids in magnetic fields. It is important role in heat and momentum transfer in apply different era like satellites, solar reactors, electronic devices, and aeronautics. Sohail et al.^5^ investigated the entropy generation in three-dimensional (3D) nanofluid flow that involves the suspension of nanoparticles and gyrotactic microorganisms with heat radiation. They used the homotopy analysis method to solve the nonlinear differential equations involved. Mallick et al.^6^ analyzed the effects of Joule heating and Hall current on the electrokinetic flow of nanofluids through a saturated porous medium, also considering entropy production. They utilized the Adomian decomposition scheme to obtain series solutions for their study. Alshomrani et al.^7^ developed a mathematical model from governing PDEs/ODEs to describe the behavior of a non-Newtonian magneto-cross nanofluid flowing over a wedge. They examined mass and heat transfer rates, activation energy and the presence of motile microbial inhabitants. Awan et al.^8^ provided a deep analysis of the magnetohydrodynamic (MHD) effects and Hall current in the flow of micropolar with the nanofluids between parallel plates.

Tripathi et al.^9^ examined how both a magnetic-field and electro-osmotic forces influence the time-dependent peristaltic flow through mathematical model in a microchannel. They focused on how these combined forces affect the movement of fluid through the channel, where peristaltic waves (wave-like contractions) drive the flow. Singh et al.^10^ studied the swimming behavior of two types of biological microswimmer populations and Venkata et al.^11^ explored the effects of magnetohydrodynamic (MHD) in Casson fluid flow over a convective surface. The investigation included factors such as cross-diffusion, chemical reactions, and non-linear radiative heat transfer. Haider et al.^12^ investigated the two-phase peristaltic flow in the presence of a magnetic field and considered the influence of wall characteristics. Pozrikidis et al.^13^ utilized the boundary integral method to examine how various physical important factors affect certain variables in peristaltic flow within 2D channels, assuming a slow or “creeping” flow. Asha and Deepa et al.^14^ investigated the entropy production in the flow of micropolar fluids through a tapered channel, taking into account thermal radiation and magnetic effects. Vishalakshi et al.^15^ explored the steady-state 2D flow of Rivlin-Ericksen magnetohydrodynamic (MHD) fluids resulting from the stretching of a porous material sheet. Hung and Brown et al.^16^ conducted an experimental study on the transfer of solid particles in peristaltic flow using a two-dimensional model. They found that when a particle’s center is not aligned with the longitudinal axis, both rotation and translation of the particle occur.

Saleem et al.^17^ presented an exact solution for the electro-osmotic particulate flow of a Jeffrey fluid. Their study highlights the influence of key parameters, providing a detailed parametric analysis. Nazeer et al.^18^ suggested that the governing equations for fluid and particle phases using continuity, momentum, and energy equations, employing long wavelength approximation and creeping flow regime. Hall current and porous medium terms were incorporated into the momentum equations to account for their effects, while thermal radiation included in the energy equation. Alqarni et al.^19^ suggested mathematical models to study the fluid and particulate phases using a lubrication approach due to laminar flow and small channel dimensions. The study utilized the NDSolve tool in Mathematica to solve a system of differential equations. The system of partial differential equations developed by Aly et al.^20^ to describe the peristaltic flow of a nanofluid with the slip effect of temperature, velocity, and It is possible to solve concentration analytically. The specific solutions that acquired used in the study effects of thermophoresis, Brownian, and the slip parameter numerous more parameters on the motion parameters temperature distribution, velocity profiles, and pressure rise gradient in pressure and concentration of nanoparticles.

The impact of Joule heat on Carreau nanofluid in peristaltic movement in an inclined asymmetric channel investigated by Kotnurkar et al.^21^ using the Multi-Step Differential Transformation Method. The appropriate non-dimensional parameters used to transform the governing equations into conventional non-linear partial differential equations. It is very difficult to find the numerical and analytical solutions to the transformed nonlinear partial differential equations due to their complexity. The challenges addressed by applying the semi-analytic Multi-Step Differential Transformation Method (Ms-DTM) to increase the accuracy of the solution. Zhou et al.^22^ investigated the idea of the differential transform. Ordinary and partial differential equations can be solved by analytically and numerically using the differential transform method (DTM). The method’s primary application concerns both linear and nonlinear initial value issues. Zaid et al.^23^ suggested the differential transform method (DTM), which is both an analytical and numerical technique for solving various differential equations. The method typically results in a series form solution. Momani et al.^24^ investigated the Taylor series formulated entirely differently using a semi-numerical analytical technique called the differential transform method. Rashidi et al.^25^ investigated the DTM and DTM-Padé, an analytical solution to a system of nonlinear ordinary differential equations with altered boundary conditions found. Since nonlinear equations lack exact solutions, an analysis done to compare analytical and numerical solutions. The fourth-order Runge–Kutta method used for numerical solutions. Mattheakis et al.^26^ created a neural network that solves differential equations using physics principles.

Now a day’s researcher used the artificial intelligence (AI) tools for solution of differential equations either partial or ordinary. Raissi et al.^27^ developed a valuable but under-utilized technique in scientific computing by differentiating neural networks with respect to their input coordinates and model parameters. This approach results in physics-informed neural networks, which designed to adhere to physical laws, symmetries, and conservation principles based on time-dependent and nonlinear partial differential equations. Guasti et al.^28^ explored using deep feed forward neural networks to solve ordinary and partial differential equations. His approach involved solving various initial and boundary value problems and comparing results from different network structures, activation functions, and minimization methods to exact solutions. Baymani et al.^29^ used artificial neural networks (ANN) to develop an approximate solution for the Navier–Stokes equations. While ANNs widely applied to partial differential equations, this study extends their use specifically to the Navier–Stokes problem. This method approximates the solution through a neural network-based function, with an error function formulated in terms of the trial solution. Many researchers used AI tools in different nonlinear and fluid dynamics problems^30–35^. The model studying in this research has significant applications in industrial and natural processes where different phases such as gas, liquid, or solid interact in a wavy or stratified manner. It plays a crucial role in oil and gas transportation through pipelines, chemical reactors, and heat exchangers. Understanding such flow patterns enhances efficiency, safety, and design optimization in multiphase fluid systems.

Traditional methods for solving the nonlinear PDEs/ODEs of multi-phase wavy flow are computationally expensive and time-consuming. The application of artificial intelligence, particularly neural networks, to solve these equations efficiently fully explored. The research gap lies in the lack of literature exploring the hybridization of two nonlinear activation functions for studying multiphase flow problems. The hybrid of nonlinear continuous activation functions has not widely used in neural networks for solving multi-phase wavy flow models. This study introduces a novel architecture called Morlet-Tanh Neural Networks (MTNNs) with MATLAB R2021a, which differs from traditional PINNs in both the activation function design and the optimization approach. While PINNs rely on standard activation functions (such as Tanh or ReLU) and gradient-based solvers, MTNNs enhancing the ability to capture complex nonlinear dynamics with higher precision. Moreover, the problem containing both the electroosmosis and magnetic field for pumping flow of Carreau model has not been discussed in the literature. Such flows have a great application in medical and industrial sectors. In this work, the proposed research fills these gaps by presenting an AI-based MTNNs to execute multi-phase wavy flow systems, generating a more accurate and efficient solution for the considered physical problem. Major outcomes of the study are described as.

1. To get more accurate and optimized solutions for the problem
2. Best fit function criteria to draw velocity and thermal graphs
3. To get the analysis of magnetic field and ionic distribution on the flow
4. To get the MTNNs solutions for nonlinear system of differential equations
5. To get dual phase flow analysis of Carreau fluid model
6. To get the impact of the compliant walls on peristaltic flow

## Formulation of problems

In this work, we examined a particulate-fluid mixture in a two-dimensional (2D) micro peristaltic channel using the non-linear Carreau fluid model. The fluid phase provided with electro-kinetic energy by attaching an external battery; however, impact of electro-osmosis on the solid phase and temperature profile didn’t take into consideration by the authors. Vertical coordinate and time designed as independent variables, and channel walls made to vibrate in response to waves that propagated horizontally Perpendicular to the flow direction, an external magnetic-field of constant strength ${B}_{0}$ applied. In mathematics, the wavy walls functions are represented as^32^.

1 $$\pm H\left(\widetilde{x},\widetilde{t}\right)=\pm \widetilde{a} \pm \widetilde{b} sin \frac{2\pi }{ \lambda } \left(\widetilde{x} - \widetilde{c}\widetilde{t}\right),$$

where $\widetilde{a}$ is the semi-width of the given channel, $\widetilde{c}$ is the speed of wave propagation $\widetilde{b}$ is the average amplitude of the wave, $\widetilde{t}$ is the wavelength, while λ is the time factor.

Mathematical form of the Carreau model is described here^19,21^

2 $${\varvec{\tau}}= {\mu }_{os}{\left(1+{\left(\Gamma \widetilde{\dot{\gamma }}\right)}^{2}\right)}^{\frac{n-1}{2}}\widetilde{\dot{{\varvec{\gamma}}}}.$$

where ${\mu }_{os}$ is zero shear rate viscosity, $\widetilde{\dot{{\varvec{\gamma}}}}$ gives the shear rate tensor, *n* is the power law index and $\Gamma$ provides the time constant. To translate the Boltzman equation, we write^6,9,17^

3 $$\frac{{\partial }^{2}\overline{\varphi }}{\partial {\widetilde{x} }^{2}}+ \frac{{\partial }^{2}\overline{\varphi }}{\partial {\widetilde{y} }^{2}}={k}^{2}\overline{\varphi }.$$

In non-dimensional form, the governing nonlinear equations for the fluid and particle phases expressed as in^32^

4 $$\frac{\partial {u}_{f,p}}{\partial x}+\frac{\partial {u}_{f,p}}{\partial y}=0,$$

5 $$\begin{aligned}\left(1-{c}_{1}\right){R}_{e}\delta \left(\frac{\partial {u}_{f}}{\partial t}+{u}_{f}\frac{\partial {u}_{f}}{\partial x}+{v}_{f}\frac{\partial {u}_{f}}{\partial y}\right)= &-\left(1-{c}_{1}\right)\frac{\partial p}{\partial x}+{c}_{1}{N}_{1}\left({u}_{p}-{u}_{f}\right)\\ & +\left(1-{c}_{1}\right)\left(\delta \frac{\partial }{\partial x}{\tau }_{xx}+\frac{\partial }{\partial y}{\tau }_{xy}\right) \\ & +\frac{{M}^{2}}{\left(1+{m}^{2}\right)}\left(m\delta {v}_{f}-{u}_{f}\right)+{U}_{e}{k}^{2}\varphi ,\end{aligned}$$

6 $$\begin{aligned}\left(1-{c}_{1}\right){\delta }^{3}Re \left(\frac{\partial {v}_{f}}{\partial t}+{u}_{f}\frac{\partial {v}_{f}}{\partial x}+{v}_{f}\frac{\partial {v}_{f}}{\partial y}\right)= & -\left(1-{c}_{1}\right)\frac{\partial p}{\partial y}+\delta \left(1-{c}_{1}\right)\left(\delta \frac{\partial {\tau }_{yx}}{\partial x}+\frac{\partial {\tau }_{yy}}{\partial y}\right) \\ & +{\delta }^{2}{c}_{1}{N}_{1}\left({v}_{p}-{v}_{f}\right) \\ & -\delta \frac{{M}^{2}}{\left(1+{m}^{2}\right)}\left(m{u}_{f}-\delta {v}_{f}\right)+\delta {U}_{e}{k}^{2}\varphi ,\end{aligned}$$

7 $$Re\left(1-{c}_{1}\right)\delta \left(\frac{\partial {\theta }_{f}}{\partial t}+{u}_{f}\frac{\partial {\theta }_{f}}{\partial x}+{v}_{f}\frac{\partial {\theta }_{f}}{\partial y}\right)=\frac{1}{{P}_{r}}\left(1-{c}_{1}\right)\frac{{\partial }^{2}{\theta }_{f}}{\partial {y}^{2}}+{c}_{1}{N}_{1}{E}_{c}{\left(\frac{dp}{dx}\frac{1}{{N}_{1}}\right)}^{2}+\left(1-{c}_{1}\right){E}_{c}{\tau }_{xy}\frac{\partial {u}_{f}}{\partial y},$$

8 $$Re\delta \left(\frac{\partial {u}_{p}}{\partial t}+{u}_{p}\frac{\partial {u}_{p}}{\partial x}+{v}_{p}\frac{\partial {u}_{p}}{\partial y}\right)+\frac{\partial p}{\partial x}={N}_{1}\left({u}_{f}-{u}_{p}\right),$$

9 $$Re{\delta }^{3}\left(\frac{\partial {v}_{p}}{\partial t}+{u}_{p}\frac{\partial {v}_{p}}{\partial x}+{\delta v}_{p}\frac{\partial {v}_{p}}{\partial y}\right)+\frac{\partial p}{\partial y}= {\delta }^{2}{N}_{1}\left({v}_{f}-{v}_{p}\right),$$

10 $$\delta \left(\frac{\partial {\theta }_{p}}{\partial t}+{u}_{p}\frac{\partial {\theta }_{p}}{\partial x}+{v}_{p}\frac{\partial {\theta }_{p}}{\partial y}\right)=\alpha \left({\theta }_{f}-{\theta }_{p}\right),$$

where ς is the zeta potential, ${U}_{e}$ the Helmholtz-Smoluchowski velocity, and ${\lambda }_{d}$ represents Debye length. The dimension-less shape of the Boltzman equation is given

11 $$\frac{{d}^{2}\varphi }{d{y}^{2}}={k}^{2}\varphi ,$$

The following dimension-less limit conditions describe it:

12 $${\left.\frac{\partial \varphi }{\partial y}\right|}_{y=0}=0\text{ and }{\left.\varphi \right|}_{y=h}=0.$$

By resolving Eq. (11) as well as applying the boundary conditions in Eq. (12), The resulting potential can be obtained as:

13 $$\varphi =\frac{\text{cosh}ky}{\text{cosh}kh}.$$

Ultimately, the following expressions were derived for the velocities and temperatures of the dual phases, incorporating the lubrication strategy in reference^32^.

14 $$\frac{1}{1-{c}_{1}}\frac{dp}{dx}=\frac{{\partial }^{2}{u}_{f}}{\partial {y}^{2}}+\frac{n-1}{2}W{e}^{2}\frac{{\partial }^{2}{u}_{f}}{\partial {y}^{2}}{\left(\frac{\partial {u}_{f}}{\partial y}\right)}^{2}-\frac{1}{1-{c}_{1}}\frac{{M}^{2}}{1+{m}^{2}}{u}_{f}+\frac{1}{1-{c}_{1}}{U}_{e}{k}^{2}\frac{cosh\left(ky\right)}{cosh\left(kh\right)},$$

15 $$\frac{{\partial }^{2}{\theta }_{f}}{\partial {y}^{2}}+{E}_{c}{P}_{r}\left[\frac{\partial {u}_{f}}{\partial y}+\frac{\left(n-1\right)}{2}W{e}^{2}{\left(\frac{\partial {u}_{f}}{\partial y}\right)}^{3}\right]\left(\frac{\partial {u}_{f}}{\partial y}\right)+\frac{{c}_{1}{E}_{c}{P}_{r}}{{N}_{1}\left(1-{c}_{1}\right)}{\left(\frac{dp}{dx}\right)}^{2}=0,$$

16 $$\frac{dp}{dx}={N}_{1}\left({u}_{f}-{u}_{p}\right),$$

17 $${\theta }_{f}={\theta }_{p}.$$

The corresponding boundary conditions of these in dimensionless form

18 $$\frac{\partial {u}_{f}}{\partial y}=0, {\theta }_{f}=0, \text{at} y=0 ,$$

19 $${u}_{f}=0, {\theta }_{f}=1, \text{at } y=h=1+\phi sin2\pi \left(x-t\right).$$

## Solution methodology: Morlet wavelet ANN based model

Artificial Intelligence (AI) especially artificial Neural Networks (ANNs) can be used to solution of differential equations (DEs) by learning the function that represents the solution.

ANNs has two major types one is supervised and unsupervised. In Supervised ANNs training the network on sample data points, where each input is a point in the DEs domain, and each output is the solution value at that point. After training, the network can predict the solution for any point within the domain. Unsupervised ANNs can also solve the DEs by training the fitness function by optimizers algorithms. There are many activation are used in ANNs each have their limitation and advantages. In this study, the tanh and morlet wavelet activation function are used, both continuous and differentiable function, so we hybrid these activations functions as Morlet-Tanh neural networks (MTNNs) for enhance the accuracy the function.

The outlines of the importance of MTNNs are.

- The MTNNs combines the localization ability of Morlet wavelets with the nonlinearity of Tanh, enhancing accuracy in solving ODEs.
- It captures both global and local features of the solution effectively.
- This hybrid activation improves convergence speed and generalization in complex differential systems.

The morlet wavelet activation function is follows:

20 $$U(x)=\sum_{i=1}^{t}\left({e}^{-0.5{\left({{\beta }_{{u}_{f}}}_{i}+{{\alpha }_{{u}_{f}}}_{i}y\right)}^{2}}\text{cos}\left(\left({{\beta }_{{u}_{f}}}_{i}+{{\alpha }_{{u}_{f}}}_{i}y\right)\right)\right),$$

The Tanh activation function is follows:

21 $$V(U(x))=\sum_{i=1}^{t}Tanh (U(x)),$$

The morlet wavelet and Tanh activation function and its derivatives are following:

22 $$\widehat{{u}_{f}(y)}=\sum_{i=1}^{t}{\gamma }_{{{u}_{f}}_{i}}Tanh\left({e}^{-0.5{\left({{\beta }_{{u}_{f}}}_{i}+{{\alpha }_{{u}_{f}}}_{i}y\right)}^{2}}\text{cos}\left(\left({{\beta }_{{u}_{f}}}_{i}+{{\alpha }_{{u}_{f}}}_{i}y\right)\right)\right),$$

23 $$\begin{aligned} \widehat{{\frac{{\partial u_{f} (y)}}{{\partial y}}}} = & \sum\limits_{{i = 1}}^{t} {\gamma _{{u_{{f_{i} }} }} } Sech\left( {e^{{ - 0.5\left( {\beta _{{u_{{f_{i} }} }} + \alpha _{{u_{{f_{i} }} }} y} \right)^{2} }} \cos \left( {\left( {\beta _{{u_{{f_{i} }} }} + \alpha _{{u_{{f_{i} }} }} y} \right)} \right)} \right)^{2} \left( { - \alpha _{{u_{{f_{i} }} }} e^{{ - 0.5\left( {\beta _{{u_{{f_{i} }} }} + \alpha _{{u_{{f_{i} }} }} y} \right)^{2} }} \left( {\beta _{{u_{{f_{i} }} }} + \alpha _{{u_{{f_{i} }} }} y} \right)\cos \left( {\left( {\beta _{{u_{{f_{i} }} }} + \alpha _{{u_{{f_{i} }} }} y} \right)} \right)} \right. \\ & \quad \left. { - \alpha _{{u_{{f_{i} }} }} e^{{ - 0.5\left( {\beta _{{u_{{f_{i} }} }} + \alpha _{{u_{{f_{i} }} }} y} \right)^{2} }} \sin \left( {\left( {\beta _{{u_{{f_{i} }} }} + \alpha _{{u_{{f_{i} }} }} y} \right)} \right)} \right), \\ \end{aligned}$$

24 $$\begin{aligned} \widehat{{\frac{{\partial ^{2} u_{f} }}{{\partial y^{2} }}}} = & \sum\limits_{{i = 1}}^{t} {\gamma _{{u_{{f_{i} }} }} } \text{Sech} \left( {e^{{ - 0.5\left( {\beta _{{u_{{f_{i} }} }} + \alpha _{{u_{{f_{i} }} }} y} \right)^{2} }} \cos \left( {\left( {\beta _{{u_{{f_{i} }} }} + \alpha _{{u_{{f_{i} }} }} y} \right)} \right)} \right)^{2} \\ & \left( { - 2\left( {\alpha _{{u_{{f_{i} }} }} } \right)^{2} e^{{ - 0.5\left( {\beta _{{u_{{f_{i} }} }} + \alpha _{{u_{{f_{i} }} }} y} \right)^{2} }} \cos \left( {\left( {\beta _{{u_{{f_{i} }} }} + \alpha _{{u_{{f_{i} }} }} y} \right)} \right)} \right. \\ & + \left( {\alpha _{{u_{{f_{i} }} }} } \right)^{2} e^{{ - 0.5\left( {\beta _{{u_{{f_{i} }} }} + \alpha _{{u_{{f_{i} }} }} y} \right)^{2} }} \left( {\beta _{{u_{{f_{i} }} }} + \alpha _{{u_{{f_{i} }} }} y} \right)^{2} \cos \left( {\left( {\beta _{{u_{{f_{i} }} }} + \alpha _{{u_{{f_{i} }} }} y} \right)} \right) \\ & \left. { + 2\left( {\alpha _{{u_{{f_{i} }} }} } \right)^{2} e^{{ - 0.5\left( {\beta _{{u_{{f_{i} }} }} + \alpha _{{u_{{f_{i} }} }} y} \right)^{2} }} \left( {\beta _{{u_{{f_{i} }} }} + \alpha _{{u_{{f_{i} }} }} y} \right)\sin \left( {\left( {\beta _{{u_{{f_{i} }} }} + \alpha _{{u_{{f_{i} }} }} y} \right)} \right)} \right) \\ & - 2\text{Sech} \left( {e^{{ - 0.5\left( {\beta _{{u_{{f_{i} }} }} + \alpha _{{u_{{f_{i} }} }} y} \right)^{2} }} \cos \left( {\left( {\beta _{{u_{{f_{i} }} }} + \alpha _{{u_{{f_{i} }} }} y} \right)} \right)} \right)^{2} \\ & \left( { - \alpha _{{u_{{f_{i} }} }} e^{{ - 0.5\left( {\beta _{{u_{{f_{i} }} }} + \alpha _{{u_{{f_{i} }} }} y} \right)^{2} }} \left( {\beta _{{u_{{f_{i} }} }} + \alpha _{{u_{{f_{i} }} }} } \right)\cos \left( {\left( {\beta _{{u_{{f_{i} }} }} + \alpha _{{u_{{f_{i} }} }} y} \right)} \right)} \right. \\ & - 2\alpha _{{u_{{f_{i} }} }} ^{{ - 0.5\left( {\beta _{{u_{{f_{i} }} }} + \alpha _{{u_{{f_{i} }} }} y} \right)^{2} }} \sin \left( {\left( {\beta _{{u_{{f_{i} }} }} + \alpha _{{u_{{f_{i} }} }} y} \right)} \right)^{2} \\ & \left. {\text{Tanh} \left( {e^{{ - 0.5\left( {\beta _{{u_{{f_{i} }} }} + \alpha _{{u_{{f_{i} }} }} y} \right)^{2} }} \cos \left( {\left( {\beta _{{u_{{f_{i} }} }} + \alpha _{{u_{{f_{i} }} }} y} \right)} \right)} \right)} \right), \\ \end{aligned}$$

25 $$\widehat{{\theta }_{f}(y)}=\sum_{i=1}^{t}{\gamma }_{{{\theta }_{f}}_{i}}Tanh\left({e}^{-0.5{\left({{\beta }_{{\theta }_{f}}}_{i}+{{\alpha }_{{\theta }_{f}}}_{i}y\right)}^{2}}\text{cos}\left(\left({{\beta }_{{\theta }_{f}}}_{i}+{{\alpha }_{{\theta }_{f}}}_{i}y\right)\right)\right),$$

26 $$\begin{aligned} \widehat{{\frac{{\partial \theta _{f} (y)}}{{\partial y}}}} = & \sum\limits_{{i = 1}}^{t} {\gamma _{{\theta _{{f_{i} }} }} } \text{Sech} \left( {e^{{ - 0.5\left( {\beta _{{\theta _{{f_{i} }} }} + \alpha _{{\theta _{{f_{i} }} }} y} \right)^{2} }} \cos \left( {\left( {\beta _{{\theta _{{f_{i} }} }} + \alpha _{{\theta _{{f_{i} }} }} y} \right)} \right)} \right)^{2} \\ & \left( { - \alpha _{{\theta _{{f_{i} }} }} e^{{ - 0.5\left( {\beta _{{\theta _{{f_{i} }} }} + \alpha _{{\theta _{{f_{i} }} }} y} \right)^{2} }} \left( {\beta _{{\theta _{{f_{i} }} }} + \alpha _{{\theta _{{f_{i} }} }} y} \right)\cos \left( {\left( {\beta _{{\theta _{{f_{i} }} }} + \alpha _{{\theta _{{f_{i} }} }} y} \right)} \right)} \right. \\ & \left. { - \alpha _{{\theta _{{f_{i} }} }} e^{{ - 0.5\left( {\beta _{{\theta _{{f_{i} }} }} + \alpha _{{\theta _{{f_{i} }} }} y} \right)^{2} }} \sin \left( {\left( {\beta _{{\theta _{{f_{i} }} }} + \alpha _{{\theta _{{f_{i} }} }} y} \right)} \right)} \right), \\ \end{aligned}$$

27 $$\begin{aligned} \widehat{\frac{{\partial }^{2}{\theta }_{f}}{\partial {y}^{2}}}= & \sum_{i=1}^{t}{\gamma }_{{{\theta }_{f}}_{i}}Sech{\left({e}^{-0.5{\left({{\beta }_{{\theta }_{f}}}_{i}+{{\alpha }_{{\theta }_{f}}}_{i}y\right)}^{2}}\text{cos}\left(\left({{\beta }_{{\theta }_{f}}}_{i}+{{\alpha }_{{\theta }_{f}}}_{i}y\right)\right)\right)}^{2} \\ & \left( { - 2\left( {\alpha _{{\theta _{{f_{i} }} }} } \right)^{2} e^{{ - 0.5\left( {\beta _{{\theta _{{f_{i} }} }} + \alpha _{{\theta _{{f_{i} }} }} y} \right)^{2} }} {\text{cos}}\left( {\left( {\beta _{{\theta _{{f_{i} }} }} + \alpha _{{\theta _{{f_{i} }} }} y} \right)} \right)} \right. \\ & + \left( {\alpha _{{\theta _{{f_{i} }} }} } \right)^{2} e^{{ - 0.5\left( {\beta _{{\theta _{{f_{i} }} }} + \alpha _{{\theta _{{f_{i} }} }} y} \right)^{2} }} \left( {\beta _{{\theta _{{f_{i} }} }} + \alpha _{{\theta _{{f_{i} }} }} y} \right)^{2} {\text{cos}}\left( {\left( {\beta _{{\theta _{{f_{i} }} }} + \alpha _{{\theta _{{f_{i} }} }} y} \right)} \right) \\ & \left. { + 2\left( {\alpha _{{\theta _{{f_{i} }} }} } \right)^{2} e^{{ - 0.5\left( {\beta _{{\theta _{{f_{i} }} }} + \alpha _{{\theta _{{f_{i} }} }} y} \right)^{2} }} \left( {\beta _{{\theta _{{f_{i} }} }} + \alpha _{{\theta _{{f_{i} }} }} y} \right){\text{sin}}\left( {\left( {\beta _{{\theta _{{f_{i} }} }} + \alpha _{{\theta _{{f_{i} }} }} y} \right)} \right)} \right) \\ & - 2Sech\left( {e^{{ - 0.5\left( {\beta _{{\theta _{{f_{i} }} }} + \alpha _{{\theta _{{f_{i} }} }} y} \right)^{2} }} {\text{cos}}\left( {\left( {\beta _{{\theta _{{f_{i} }} }} + \alpha _{{\theta _{{f_{i} }} }} y} \right)} \right)} \right)^{2} \\ & \left( { - \alpha _{{\theta _{{f_{i} }} }} e^{{ - 0.5\left( {\beta _{{\theta _{{f_{i} }} }} + \alpha _{{\theta _{{f_{i} }} }} y} \right)^{2} }} \left( {\beta _{{\theta _{{f_{i} }} }} + \alpha _{{\theta _{{f_{i} }} }} } \right){\text{cos}}\left( {\left( {\beta _{{\theta _{{f_{i} }} }} + \alpha _{{\theta _{{f_{i} }} }} y} \right)} \right)} \right. \\ & - \alpha _{{\theta _{{f_{i} }} }} e^{{ - 0.5\left( {\beta _{{\theta _{{f_{i} }} }} + \alpha _{{\theta _{{f_{i} }} }} y} \right)^{2} }} \left( {\beta _{{\theta _{{f_{i} }} }} + \alpha _{{\theta _{{f_{i} }} }} y} \right){\text{cos}}\left( {\left( {\beta _{{\theta _{{f_{i} }} }} + \alpha _{{\theta _{{f_{i} }} }} y} \right)} \right) \\ & - 2\alpha _{{\theta _{{f_{i} }} }} e^{{ - 0.5\left( {\beta _{{\theta _{{f_{i} }} }} + \alpha _{{\theta _{{f_{i} }} }} y} \right)^{2} }} {\text{sin}}\left( {\left( {\beta _{{\theta _{{f_{i} }} }} + \alpha _{{\theta _{{f_{i} }} }} y} \right)} \right)^{2} \\ & \left. {Tanh\left( {e^{{ - 0.5\left( {\beta _{{\theta _{{f_{i} }} }} + \alpha _{{\theta _{{f_{i} }} }} y} \right)^{2} }} {\text{cos}}\left( {\left( {\beta _{{\theta _{{f_{i} }} }} + \alpha _{{\theta _{{f_{i} }} }} y} \right)} \right)} \right)} \right). \\ \end{aligned}$$

where $v(U\left(x\right))$ symbolizes a function of activation. The velocity and temperature equation expressions become when the feed-forward ANNs used.

28 $$\frac{1}{1-C}\frac{dp}{dx}=\frac{{d}^{2}\widehat{{u}_{f}(y)}}{{dy}^{2}}+\frac{n-1}{2}W{e}^{2}\frac{{d}^{2}\widehat{{u}_{f}(y)}}{{dy}^{2}}{\left(\frac{d\widehat{{u}_{f}(y)}}{dy}\right)}^{2}-\frac{1}{1-C}\frac{{M}^{2}}{1+{m}^{2}}\widehat{{u}_{f}\left(y\right)}+\frac{1}{1-C}{U}_{e}{k}^{2}\frac{cosh\left(ky\right)}{cosh\left(kh\right)},$$

29 $$\frac{{d}^{2}\widehat{\theta (y)}}{{dy}^{2}}+{E}_{c}{P}_{r}\left[\frac{d\widehat{{u}_{f}(y)}}{dy}+\frac{\left(n-1\right)}{2}W{e}^{2}{\left(\frac{d\widehat{{u}_{f}(y)}}{dy}\right)}^{3}\right]\left(\frac{d\widehat{{u}_{f}(y)}}{dy}\right)+\frac{C{E}_{c}{P}_{r}}{{N}_{1}\left(1-C\right)}{\left(\frac{dp}{dx}\right)}^{2}=0.$$

The hyperparameters (weights and biases) are fundamental components of MTNNs that determine how input signals are transformed through layers. They control the strength and direction of signal flow, enabling the network to learn patterns from data through optimization. Proper tuning of weights and biases is essential for achieving high accuracy and generalization in predictions. The approximate solution by Eqs. (28) and (29) using set $$W=60$$, which represents the weights of 90 matching Morlet-Tanh neural networks (MTNNs). $$W=\left[\alpha {{u}_{f}}_{i}, \beta {{u}_{f}}_{i},{{\gamma }_{{u}_{f}}}_{i},\alpha {{\theta }_{f}}_{i}, \beta {{\theta }_{f}}_{i},{{\gamma }_{{\theta }_{f}}}_{i}\right]$$, as well as its components are $$\alpha u_{{f_{i} }} = \{ \alpha u_{{f1}} ,$$$$\alpha {{u}_{f}}_{2},\alpha {{u}_{f}}_{3},$$$$\dots ,\alpha {{u}_{f}}_{10}\},$$$$\beta {{u}_{f}}_{i}= \{\beta {{u}_{f}}_{1},$$$$\beta u_{{f_{2} }} ,\beta u_{{f_{3} }} ,$$$$\ldots ,\beta u_{{f_{{10}} }} \},$$ $$\gamma {{u}_{f}}_{i}=$$$$\{\gamma {{u}_{f}}_{1},$$$$\gamma {{u}_{f}}_{2},$$$$\gamma u_{{f_{3} }} , \ldots ,\gamma u_{{f_{{10}} }} \} ,$$$$\alpha {{\theta }_{f}}_{i}=\{\alpha {{\theta }_{f}}_{1},$$$$\alpha {{\theta }_{f}}_{2},\alpha {{\theta }_{f}}_{3},$$$$\dots ,\alpha {{\theta }_{f}}_{10}\},$$$$\beta {{\theta }_{f}}_{i}=\{\beta {{\theta }_{f}}_{1},$$$$\beta {{\theta }_{f}}_{2},\beta {{\theta }_{f}}_{3},\dots ,$$$$\beta \theta _{{f_{{10}} }} \} ,\gamma \theta _{{f_{i} }} =$$$$\{ \gamma \theta _{{f_{1} }} ,\gamma \theta _{{f_{2} }} ,$$$$\gamma \theta _{{f_{3} }} , \ldots ,\gamma \theta _{{f_{{10}} }} \}$$ that optimize through meta- heuristic particle swarm algorithm. The following equations represent the MTNNs-based fitness function similar to^32^.

30 $$\begin{aligned} \varepsilon _{{\widehat{{u_{f} (y)}}}} = & \sum\limits_{{i = 1}}^{t} {\left( {\frac{{\partial ^{2} \widehat{{u_{f} (y)}}}}{{\partial y^{2} }} + \frac{{n - 1}}{2}We^{2} \frac{{\partial ^{2} \widehat{{u_{f} (y)}}}}{{\partial y^{2} }}\left( {\frac{{\partial \widehat{{u_{f} (y)}}}}{{\partial y}}} \right)^{2} } \right.} \\ & \quad \left. { - \frac{1}{{1 - c_{1} }}\frac{{M^{2} }}{{1 + m^{2} }}\widehat{{u_{f} (y)}} + \frac{1}{{1 - c_{1} }}U_{e} k^{2} \frac{{cosh\left( {ky} \right)}}{{cosh\left( {kh} \right)}} - \frac{1}{{1 - c_{1} }}\frac{{dp}}{{dx}}} \right)^{2} , \\ \end{aligned}$$

31 $$\begin{aligned} \epsilon _{{\widehat{{\theta _{f} (y)}}}} = & \frac{1}{{11}}\sum\limits_{{i = 1}}^{t} {\left( {\frac{{\partial ^{2} \widehat{{\hat{\theta }_{f} (y)}}}}{{\partial y^{2} }} + } \right.} \\ & \quad E_{c} P_{r} \left[ {\frac{{\partial \widehat{{u_{f} (y))arm\;and\;water\;cycle\;algorithmlgorithmynamics\;problems.}}}}{{\partial y}}} \right. \\ & \left. { + \frac{{\left( {n - 1} \right)}}{2}We^{2} \left( {\frac{{\partial \widehat{{u_{f} (y)}}}}{{\partial y}}} \right)^{3} } \right] \\ & \quad \left. {\left( {\frac{{\partial \widehat{{u_{f} (y)}}}}{{\partial y}}} \right) + \frac{{c_{1} E_{c} P_{r} }}{{N_{1} \left( {1 - c_{1} } \right)}}\left( {\frac{{dp}}{{dx}}} \right)^{2} } \right)^{2} , \\ \end{aligned}$$

32 $${\epsilon }_{\widehat{ic}}=\frac{1}{4}\left({\left(\frac{\partial \widehat{{u}_{f}(0)}}{\partial y}-0\right)}^{2}+{\left(\widehat{{\widehat{\theta }}_{f}(0)}-0\right)}^{2}+{\left(\widehat{{u}_{f}}(1+\varphi sin2\pi (x-t)\right)}^{2}+{\left({\widehat{\theta }}_{f}(1+\varphi sin2\pi \left(x-t\right)-1\right)}^{2}\right),$$

33 $$\epsilon ={\varepsilon }_{\widehat{{u}_{f}(y)}}+{\epsilon }_{\widehat{{\theta }_{f}(y)}}+{\epsilon }_{\widehat{ic}}.$$

An operator that represents a stretched membrane and a viscous damping force is shown below, i.e.

34 $$L=M\frac{{\partial }^{2}}{\partial {t}^{2}}-T\frac{{\partial }^{2}}{\partial {x}^{2}}+D\frac{\partial }{\partial t},$$

In the above equation, *M* is the mass per unit area, *D* is the coefficient of the viscous damping membrane and *T* is the elastic tension in the membrane.

35 $$\frac{ \partial p}{ \partial x}={E}_{1}\frac{{\partial }^{3}\chi }{\partial {x}^{3}}+{E}_{2}\frac{{\partial }^{3}\chi }{\partial {t}^{2}\partial x}+{E}_{3}\frac{{\partial }^{2}\chi }{\partial t\partial x} at y=1y\pm \chi$$

In the given equation, $\chi = \varphi sin 2\pi (x-t)$. The dimensionless elasticity parameters ${E}_{1},{ E}_{2}, {E}_{3}$ are defined as:

36 $$\begin{aligned}{E}_{1}=-\frac{T{\widetilde{a}}^{3}}{\widetilde{c}{\lambda }^{3}{\mu }_{s}}, \\& \quad {E}_{2}=\frac{M{\widetilde{a}}^{3}\widetilde{c}}{{\lambda }^{3}{\mu }_{s}}, \\& {E}_{3}=\frac{D{\widetilde{a}}^{3}}{{\lambda }^{2}{\mu }_{s}}. \end{aligned}$$

## Metaheuristic optimization techniques

Recent advancements in metaheuristic optimization have introduced innovative algorithms inspired by natural and mathematical phenomena. Notable examples include the Black Kite Algorithm, which uses Cauchy mutation and leadership strategies to optimize exploration and exploitation; the Secretary Bird Optimization Algorithm, based on the unique hunting behaviors of secretary birds; and the Black Eagle Optimizer, which simulates the cooperative hunting strategies of eagles, Water cycle, Particle Swarm and many others.

### Particle swarm optimization (PSO)

Evolutionary optimization system that inspired by the behavior of birds in a swarm is called PSO. PSO assists in modifying the locations of particles developed through iterative phases using both individual and global optimal solutions found by the swarm. Like a flock of birds adapting to its surroundings, PSO works by supporting a population of particles, each of which signifies a possible resolution to the optimization problem. How a particle moves to its ideal position determined by its personal best (${P}_{LB}^{x-1}$), which is its best-performing location, and its global best (${P}_{GB}^{x-1}$), which is its best-performing position as a whole. Particles interact with one another in an indirect manner. PSO has been essential in power systems for optimizing load management and energy distribution. When dealing with intricate scheduling issues such as job shop scheduling, where jobs need to be assigned PSO has demonstrated its abilities effectively with little resources. This balance makes sure that the algorithm takes advantage of the promising regions found throughout the exploration process in addition to fully exploring the solution space. This quality is very helpful in complex optimization scenarios where the terrain can be rough and complicated. PSO used by researchers to solve several difficult non-linear problems, every iteration starts with randomly selected particles and changes the particle locations and velocities according to their most recent best

37 $${v}_{i}^{x}={wv}_{i}^{t-1}+{c}_{1}{r}_{1}\left({P}_{LB}^{x-1}-{X}_{i}^{x-1} \right)+{c}_{2}{r}_{2 }\left({P}_{GB}^{x-1}-{X}_{i}^{x-1}\right)$$

Particle position

38 $${X}_{i}^{x}={X}_{i}^{x-1}+{v}_{i}^{x-1}$$

The variable *i* in these equations spans from 1 to *p*, where *p* is the total particle count. where ${v}_{i}$ is the particle’s vector of velocity and ${X}_{i}$ denotes its position within the swarm. The architecture includes weights that decrease linearly from local and global social acceleration constants (${c}_{1}$ and ${c}_{2}$), and inertia weights. The random vectors (${r}_{1}$ and ${r}_{2}$) limited inside.

## Numerical results and discussion

**Fig. 1:**
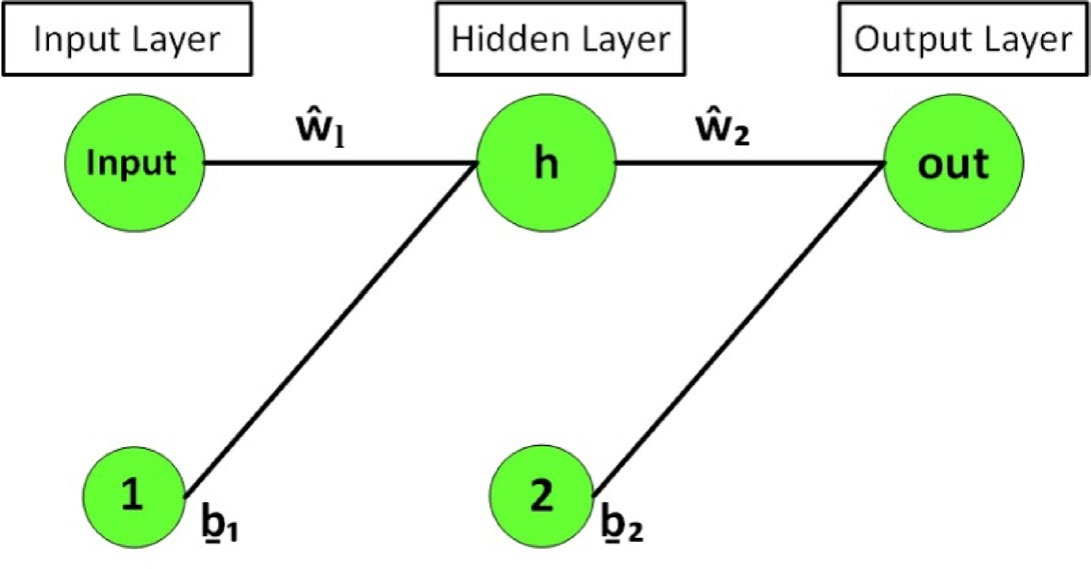
The ANN structure.

**Fig. 2:**
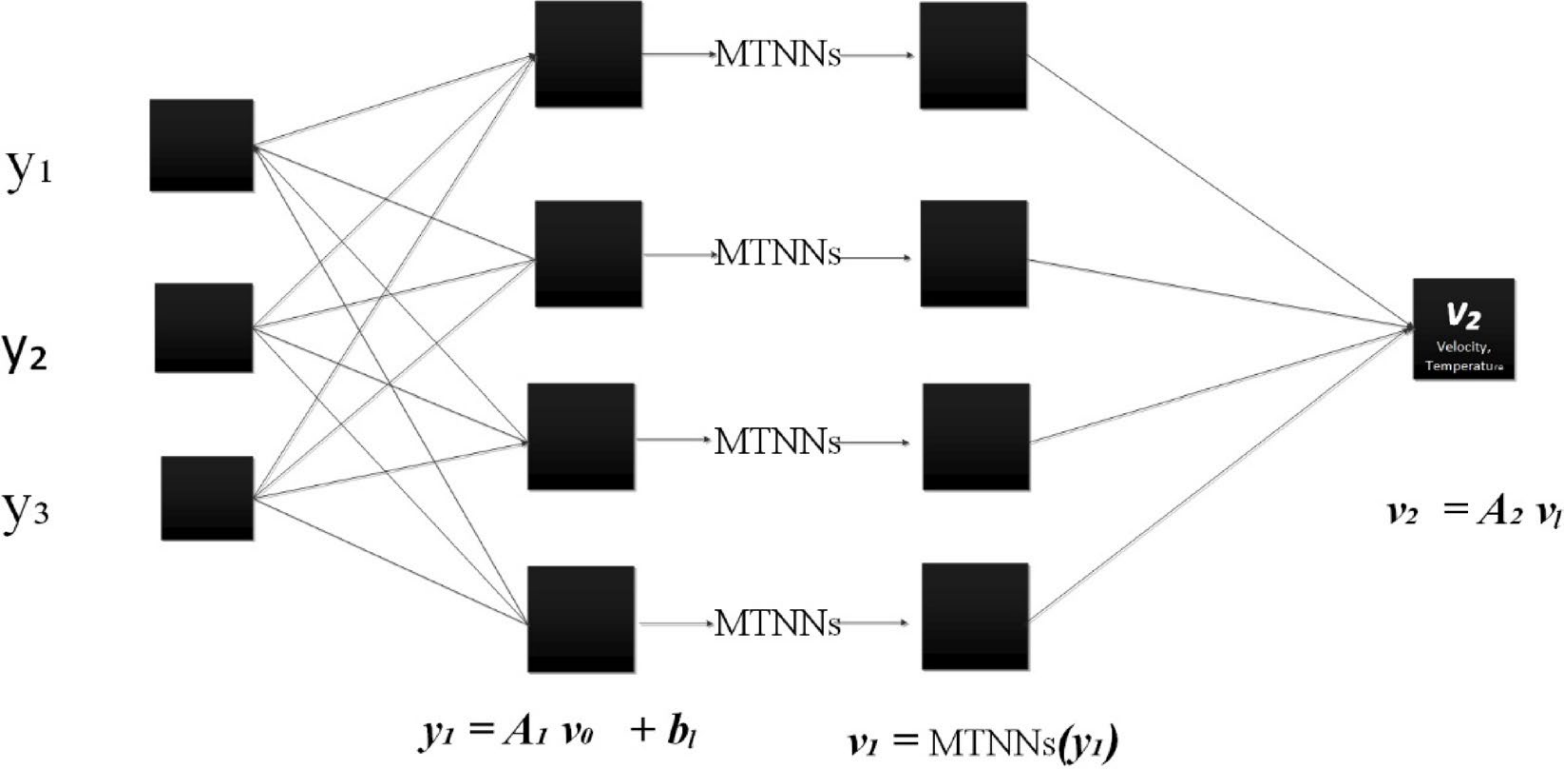
The MTNNs structure.

**Fig. 3:**
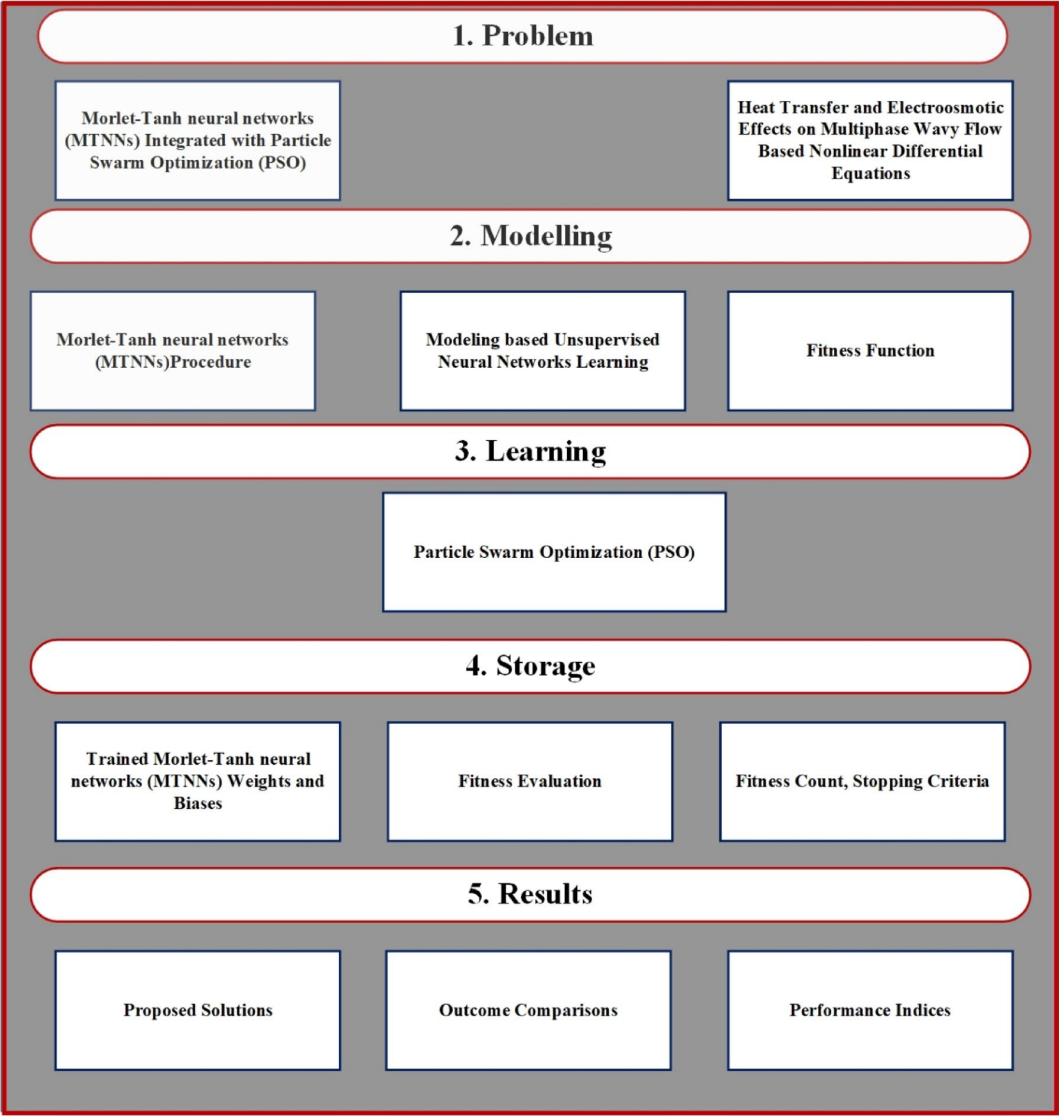
Proposed Design Methodology of MTNNs.

**Fig. 4:**
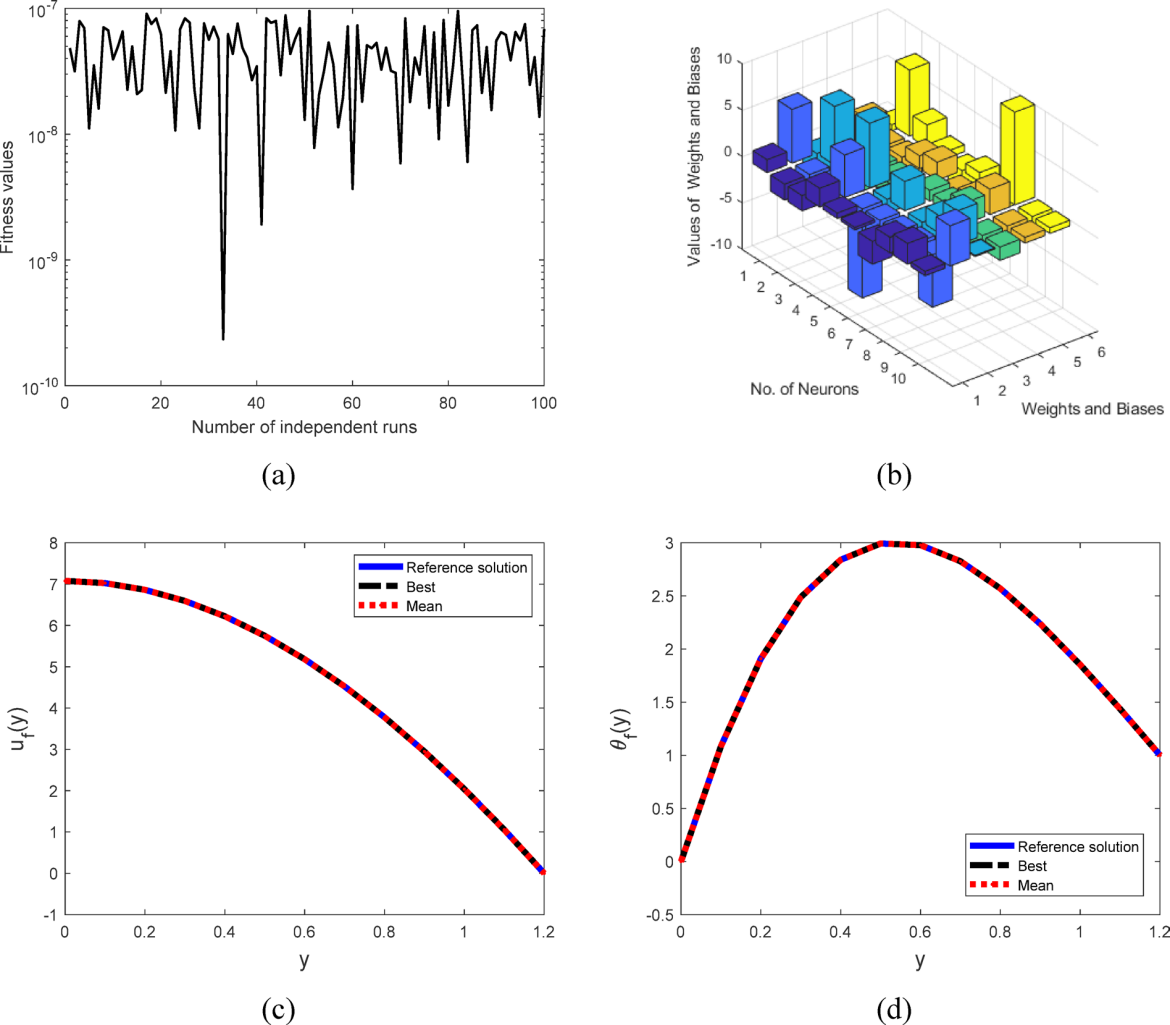
(**a**) MTNNs based fitness values across 100 independent runs, (**b**) Optimized weights through PSO ranging from −10 to 10 for $$We=0.01, h=0.941221,$$$$m=0.2, n=2,$$$${P}_{r}=2, \phi =0.1,$$$${E}_{1}=0.2,$$$${E}_{2}=0.1,$$$${E}_{3}=0.8, Ec=0.2,$$$$t=0.2, {c}_{1}=0.3,$$$${N}_{1}=2, k=2 \; \text{and} \; Ue=-1$$, (**c**). Comparison between the reference, MTNNs based best and MTNNs based mean solutions for velocity (**d**). Comparison between the reference, MTNNs based best and MTNNs based mean solutions for temperature.

The MTNNs is utilized to obtain the results of the nonlinear ODEs. The system of ODEs transformed into MTNNs based error/fitness function using sallow neural networks. The error/fitness function minimized through hybrid of particle swarm, the design ANN, MTNNs and design methodology are represented in Figs. 1, 2 and 3. The initial weights are selected as randomly between − 10 to 10, then optimize through PSO. For validation of the MTNNs methodology the obtained results compare with reference, best obtained from MTNNs and MTNNs mean solutions for fixed parameters $$We=0.01,$$$$M=0.5,$$$$k=2,$$$$m=0.1,$$$$n=2,$$$$\boldsymbol{ h}=0.941221,$$$$\phi =0.1,$$$${E}_{1}=0.2,$$$${E}_{2}=0.2,$$$${E}_{3}=0.8,$$$${P}_{r}=2,$$$$Ec=0.2,$$$$t=0.2,$$$$c1=0.3,$$$${N}_{1}=2,$$$$Ue=-1$$, represented in Fig. 4c,d, and Table 1. The optimized weights via PSO for fixed parameters are presented in Fig. 4b. To use the optimized weights in MTNNs function the solution of velocity and temperature equations represented in Eq. 46–47 for solution. To check the efficient and validation of the MTNNs methods computed the multiple runs for fitness values, all the data set belongs to zero and this scenario represents in Fig. 4a.

**Table 1:** Comparison between MTNNs and reference solution for velocity and temperature for $$We=0.01,$$$$h=0.941221,$$$$c1=0.3, M=0.5,$$$$m=0.1, n=2,$$$$Ec=0.2, \phi =0.1,$$$${E}_{1}=0.2, {E}_{2}=0.2,$$$${E}_{3}=0.8, {P}_{r}=2,$$$$t=0.2, {N}_{1}=2 ,$$$$k=2 \; \text{and} \; Ue=-1.$$

y	${u}_{f}(y)$ Reference	$\widehat{{u}_{f}(y)}$	$AE$	${\theta }_{f}\left(y\right)$ Reference	$\widehat{{\theta }_{f}(y)}$	$AE$
0	7.079224085	7.07949218442286	2.68E−04	0	-0.00012527925310	1.25E−04
0.1	7.024900857	7.02514740859084	2.47E−04	1.084842101	1.08469751258686	1.45E−04
0.2	6.862559573	6.86277164222303	2.12E−04	1.909730332	1.90962663819805	1.04E−04
0.3	6.594015567	6.59421100768277	1.95E−04	2.487935462	2.48787281876198	6.26E−05
0.4	6.222081959	6.22225010768145	1.68E−04	2.840313193	2.84028477476149	2.84E−05
0.5	5.750311644	5.75043992020039	1.28E−04	2.993590221	2.99355650455027	3.37E−05
0.6	5.182727096	5.18282967419210	1.03E−04	2.978280439	2.97829195643360	1.15E−05
0.7	4.523580625	4.52367482897407	9.42E−05	2.826461493	2.82656383894727	1.02E−04
0.8	3.777166421	3.77724317972967	7.68E−05	2.569617655	2.56973184330517	1.14E−04
0.9	2.947687714	2.94772774386873	4.00E−05	2.236697974	2.23675156174824	5.36E−05
1	2.039171948	2.03918412203109	1.22E−05	1.852461954	1.85255482396443	9.29E−05
1.1	1.05542305	1.05543139705854	8.35E−06	1.436096966	1.43632680504905	2.30E−04
1.2	7.33887E−09	−1.6144400383E−05	1.62E−05	0.999999997	1.00018019711934	1.80E−04

**Fig. 5:**
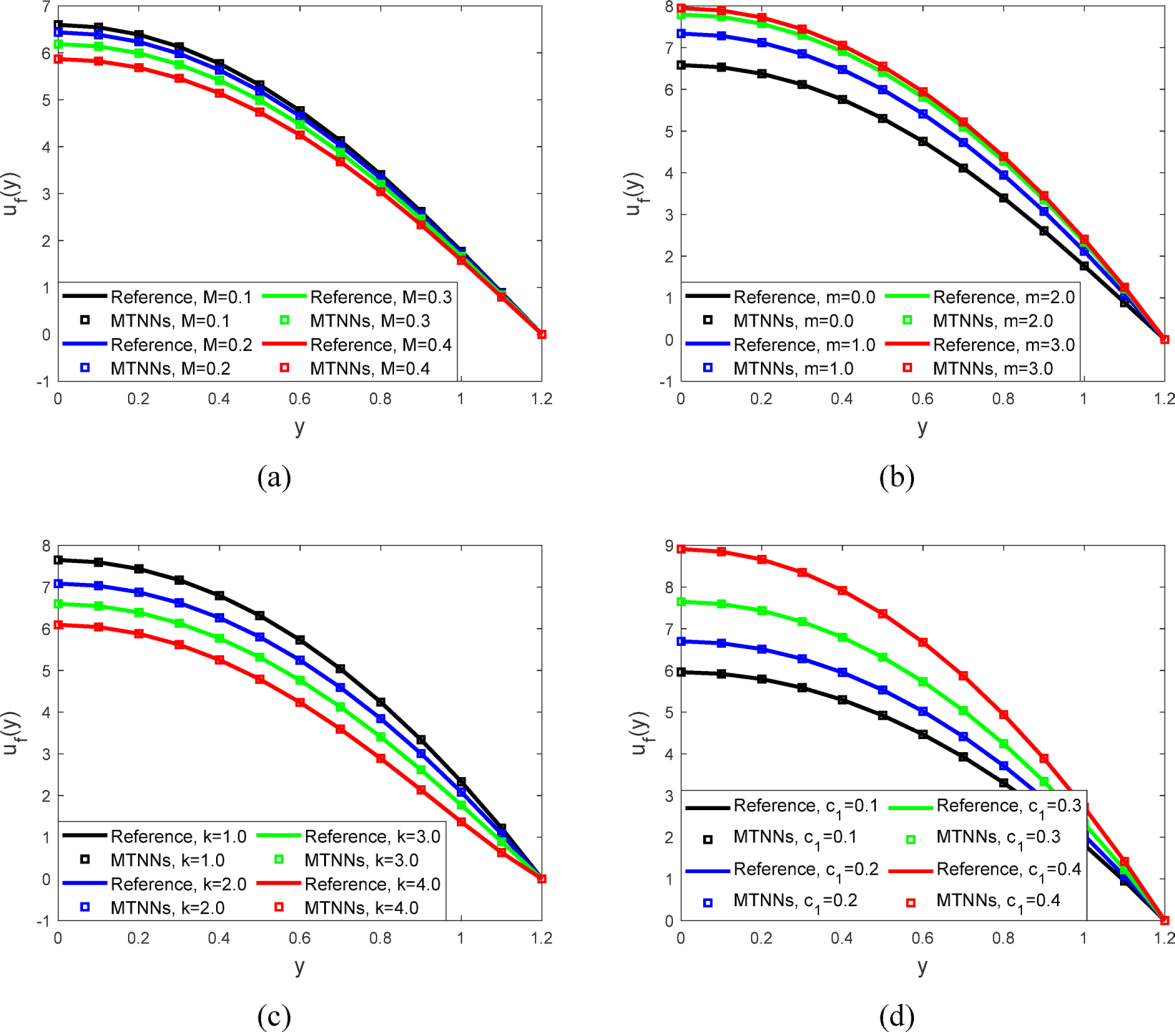
(**a**) Velocity profiles for $$h=0.941221, We=0.01,$$$$m=0.2, n=2, \phi =0.1,$$$${E}_{1}=0.2, {E}_{2}=0.1,$$$${P}_{r}=2, {E}_{3}=0.8,$$$${c}_{1}=0.3, Ec=0.2, t=0.2,$$$${N}_{1}=2, Ue=-1 \; \text{and} \; k=2.$$ (**b**). Velocity profiles for $${c}_{1}=0.3, Ec=0.2,$$$$We=0.01 ,M=0.5, n=2, \phi =0.1,$$$${E}_{1}=0.2, {E}_{2}=0.1,$$$${E}_{3}=0.8, {P}_{r}=2, t=0.2,$$$${N}_{1}=2, Ue=-1,$$$$h=0.941221, \; \text{and} \; k=2.$$ (**c**). Velocity profiles for $${P}_{r}=2, We=0.01 ,$$$${E}_{3}=0.8, M=0.5,m=0.2 n=2,$$$$\phi =0.1, {E}_{2}=0.1,$$$$Ec=0.2, t=0.2, {c}_{1}=0.3,$$$${{E}_{1}=0.2, N}_{1}=2, Ue=-1 h=0.941221.$$ (**d**). Velocity profiles for $${P}_{r}=2, We=0.01 ,$$$$M=0.5,m=0.2 n=2,$$$$h=0.941221, \phi =0.1,$$$${{E}_{3}=0.8, E}_{1}=0.2, {E}_{2}=0.1,$$$$Ec=0.2, t=0.2, {N}_{1}=2, Ue=-1 \; \text{and} \; k=2.$$

Figure 5a represented the velocity profiles against the magnetic field parameter. When the value of the magnetic field parameter (${\varvec{M}}$) increases, the flow velocity decreases. This is due to the magnetic field creating a resistive force that slows down the fluid. Thus, higher values of (${\varvec{M}}$) result in reduced flow velocity. Figure 5b showed the velocity profiles. When the value of the parameter (${\varvec{m}}$) decreases, the flow velocity increases. This is because a lower mmm reduces the resistance to fluid movement, allowing the fluid to flow more freely. Consequently, decreasing $({\varvec{m}}$) leads to higher flow velocities in the system. Figure 5c illustrated the velocity profiles the value of the parameter (${\varvec{k}}$) increases, the flow velocity decreases. This is because a higher (${\varvec{k}}$) value introduces more resistance to the fluid, slowing down the flow. Therefore, increasing (${\varvec{k}}$) results in reduced fluid velocity. Figure 5d visualized the velocity profiles, when the value of the parameter (${{\varvec{c}}}_{1}$) decreases, the flow velocity increases. A lower (${{\varvec{c}}}_{1}$) reduces resistance, allowing the fluid to flow more easily, leading to higher velocities (Tables 2 and 3).

**Table 2:** Statistical performance for multiple runs for velocity profile^32^.

$x$	Min.	Max.	Average	S.D
0	3.91E−05	2.06E−04	1.23E−04	9.43E−04
0.1	8.35E−06	4.40E−04	2.24E−04	2.69E−04
0.2	1.73E−05	1.00E−03	5.09E−04	5.48E−04
0.3	8.25E−05	3.75E−04	2.29E−04	7.38E−04
0.4	3.99E−05	5.30E−04	2.85E−04	7.01E−04
0.5	5.27E−05	3.92E−04	2.22E−04	6.66E−04
0.6	4.17E−05	9.93E−04	5.17E−04	5.39E−04
0.7	8.31E−05	1.89E−04	1.36E−04	4.18E−04
0.8	4.32E−05	6.71E−04	3.57E−04	5.27E−05
0.9	6.05E−06	2.04E−04	1.05E−04	3.01E−04
1	8.03E−05	4.53E−04	2.67E−04	9.83E−04
1.1	6.57E−05	9.86E−04	5.26E−04	6.98E−04
1.2	1.33E−05	2.88E−05	2.11E−05	4.23E−04

**Table 3:** Statistical performance for multiple runs for temperature profile^32^.

$x$	Max.	Min.	Average	S.D
0	1.56E−05	3.06E−04	1.61E−04	5.94E−04
0.1	8.56E−05	7.03E−04	3.94E−04	2.25E−05
0.2	5.83E−05	8.76E−04	4.67E−04	7.00E−04
0.3	6.45E−05	3.30E−04	1.97E−04	4.25E−04
0.4	3.85E−05	9.18E−04	4.78E−04	6.96E−04
0.5	4.28E−05	7.73E−04	4.08E−04	1.79E−04
0.6	3.76E−05	8.62E−04	4.50E−04	3.13E−04
0.7	2.26E−05	9.29E−04	4.76E−04	4.71E−04
0.8	2.52E−05	2.86E−04	1.56E−04	6.39E−04
0.9	1.91E−05	1.00E−03	5.10E−04	1.61E−04
1	1.21E−05	5.96E−04	3.04E−04	9.42E−05
1.1	4.82E−05	3.92E−04	2.20E−04	4.23E−04
1.2	5.90E−05	1.67E−04	1.13E−04	5.99E−04

**Fig. 6:**
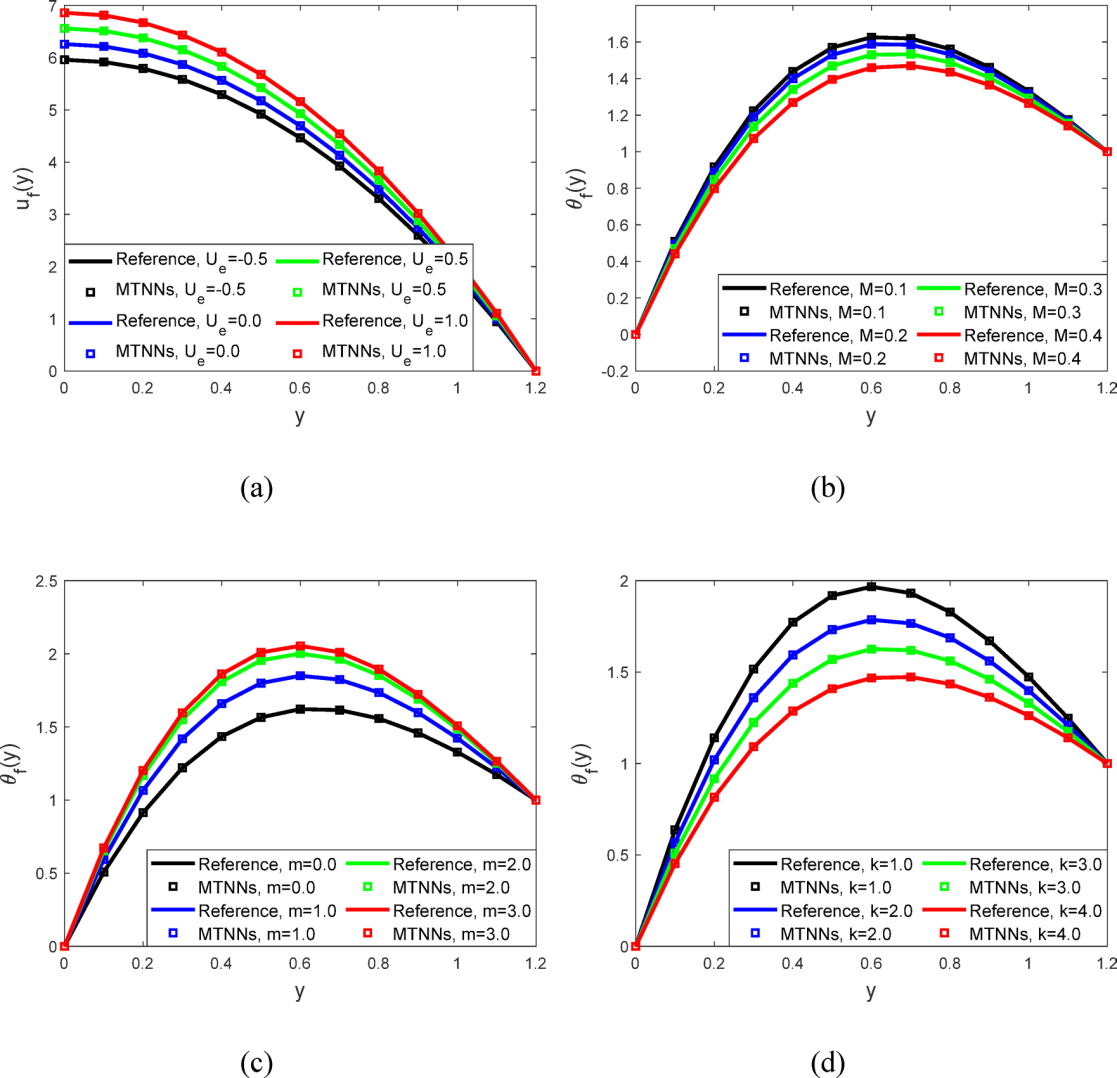
(**a**). Velocity profiles for $$t=0.2,$$$$We=0.01 ,$$$$M=0.5,$$$${E}_{2}=0.1,m=0.2,$$$${E}_{3}=0.8 n=2, h=0.941221,$$$$\phi =0.1, {E}_{1}=0.2,$$$$Ec=0.2, {P}_{r}=2,$$$${c}_{1}=0.3, {N}_{1}=2,$$$$\; \text{and} \; k=2.$$ (**b**). Temperature profiles for $${P}_{r}=2, We=0.01 ,m=0.2,$$$$Ue=-1, n=2, h=0.941221,$$$$\phi = 0.1,E_{1} = 0.2,E_{2} = 0.1,$$$${E}_{3}=0.8, Ec=0.2,$$$$t=0.2, {c}_{1}=0.3,$$$${N}_{1}=2 \; \text{and} \; k=2.$$ (**c**). Temperature profiles for $$Ec=0.2, h=0.941221, We=0.01 ,$$$$M=0.5,n=2, t=0.2,$$$$\phi =0.1, {E}_{1}=0.2,$$$${E}_{2}=0.1, {E}_{3}=0.8,$$$${P}_{r}=2, {c}_{1}=0.3, {N}_{1}=2,$$$$Ue=-1 \; \text{and} \; k=2.$$ (**d**). Temperature profiles for $$t=0.2, We=0.01 ,Ec=0.2,$$$$M=0.5,{E}_{1}=0.2,m=0.2,$$$$n=2, h=0.941221, \phi =0.1,$$$${E}_{2}=0.1, {E}_{3}=0.8,$$$${P}_{r}=2, {c}_{1}=0.3, {N}_{1}=2, Ue=-1.$$

**Fig. 7:**
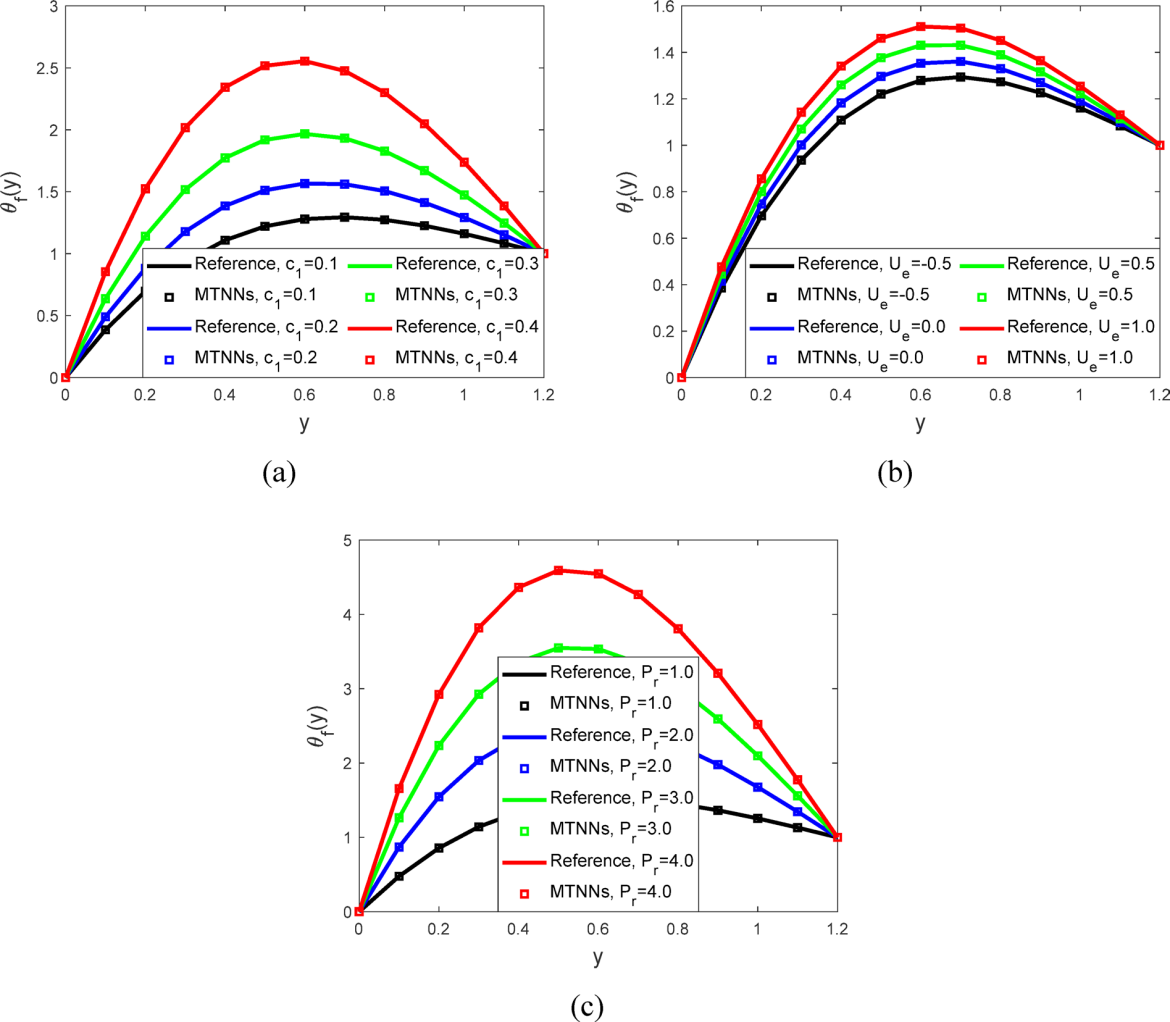
(**a**) Temperature profiles for $${P}_{r}=2, We=0.01 ,M=0.5,$$$${E}_{3}=0.8,m=0.2,n=2, h=0.941221,$$$$\phi =0.1, {E}_{1}=0.2, {E}_{2}=0.1,$$$$Ec=0.2, t=0.2, {N}_{1}=2,$$$$Ue=-1 \; \text{and} \; k=2.$$ (**b**). Temperature profiles for $${E}_{2}=0.1,We=0.01 ,M=0.5,$$$${P}_{r}=2,m=0.2,n=2,$$$$h=0.941221, \phi =0.1,$$$${E}_{1}=0.2, {E}_{3}=0.8,$$$$Ec=0.2, t=0.2,$$$${N}_{1}=2 \; \text{and} \; k=2.$$ (**c**). Temperature profiles for $${E}_{1}=0.2, We=0.01 ,h=0.941221,$$$$M=0.5,m=0.2,n=2, \phi =0.1,$$$${E}_{2}=0.1, Ec=0.2, Ue=-1 \; \text{and} \; k=2.$$$$t=0.2, {{E}_{3}=0.8, N}_{1}=2,$$$$Ue=-1 \; \text{and} \; k=2.$$

Figure 6a represented the velocity profiles. When the value of the parameter (${\varvec{U}}{\varvec{e}}$)​ (representing the velocity of the moving boundary or wall) decreases, the flow velocity increases. This is because a lower (${\varvec{U}}{\varvec{e}}$ ​) reduces the opposing effect on the fluid, allowing it to move faster within the channel. Figure 6b illustrated the temperature profiles. As the magnetic field parameter (${\varvec{M}}$) increases, the fluid temperature decreases. This occurs because the magnetic field increases the thermal conductivity of the fluid, enhancing heat transfer and allowing more heat to dissipate. As a result, the fluid cools down with higher values of (${\varvec{M}}$). Figure 6c shows the temperature profiles. When the value of the parameter (${\varvec{m}}$) decreases, the temperature of the fluid increases. This occurs because a lower (${\varvec{m}}$) reduces the fluid’s ability to dissipate heat, leading to greater thermal retention. As a result, the overall temperature of the fluid rises when (${\varvec{m}}$) is decreased. Figure 6d showed the temperature profiles for peristaltic flow. As the value of the parameter (${\varvec{k}}$) increases, the fluid temperature decreases. This occurs because a higher (k) enhances the heat transfer within the fluid, facilitating better cooling and allowing heat to dissipate more effectively, the increase in (${\varvec{k}}$) leads to a reduction in temperature. Figure 7a showed the temperature profiles with the parameter ${({\varvec{c}}}_{1})$ decreases, the temperature of the fluid increases. This is due to reduced thermal conductivity which limits heat dissipation and causes the fluid to retain more heat. Figure 7b displayed the temperature profiles with the effect parameter (${\varvec{U}}{\varvec{e}}$) ​decreases, the temperature of the fluid increases. This occurs because a lower (${\varvec{U}}{\varvec{e}}$) reduces the fluid’s motion, leading to less heat being carried away and resulting in a higher thermal accumulation within the fluid. Figure 7c illustrated the temperature profiles with physical parameter Prandtl number (${{\varvec{P}}}_{{\varvec{r}}}$) decreases, the fluid temperature increases. This occurs because a lower Prandtl number indicates a lower thermal diffusivity relative to momentum diffusivity, leading to less efficient heat transfer within the fluid. As a result, the fluid retains more heat, causing an increase in temperature. Parameter values used in graphical analysis through MTNNs tabulated in Table 4.

**Table 4:** Parameter values used in graphical analysis through MTNNs.

Profile	Parameters	Value(s) used
Velocity profile	$M$	0.1, 0.2, 0.3, 0.4
	$m$	0.0, 1.0, 2.0, 3.0
	$k$	1.0, 2.0, 3.0,4.0
	${c}_{1}$	0.1, 0.2, 0.3, 0.4
	${U}_{e}$	−0.5, 0.0, 0.5, 1.0
Temperature profile	$M$	0.1, 0.2, 0.3, 0.4
	$m$	0.0, 1.0, 2.0, 3.0
	$k$	1.0, 2.0, 3.0,4.0
	${c}_{1}$	0.1, 0.2, 0.3, 0.4
	${U}_{e}$	−0.5, 0.0, 0.5, 1.0
	${P}_{r}$	1.0, 2.0, 3.0,4.0

**Fig. 8:**
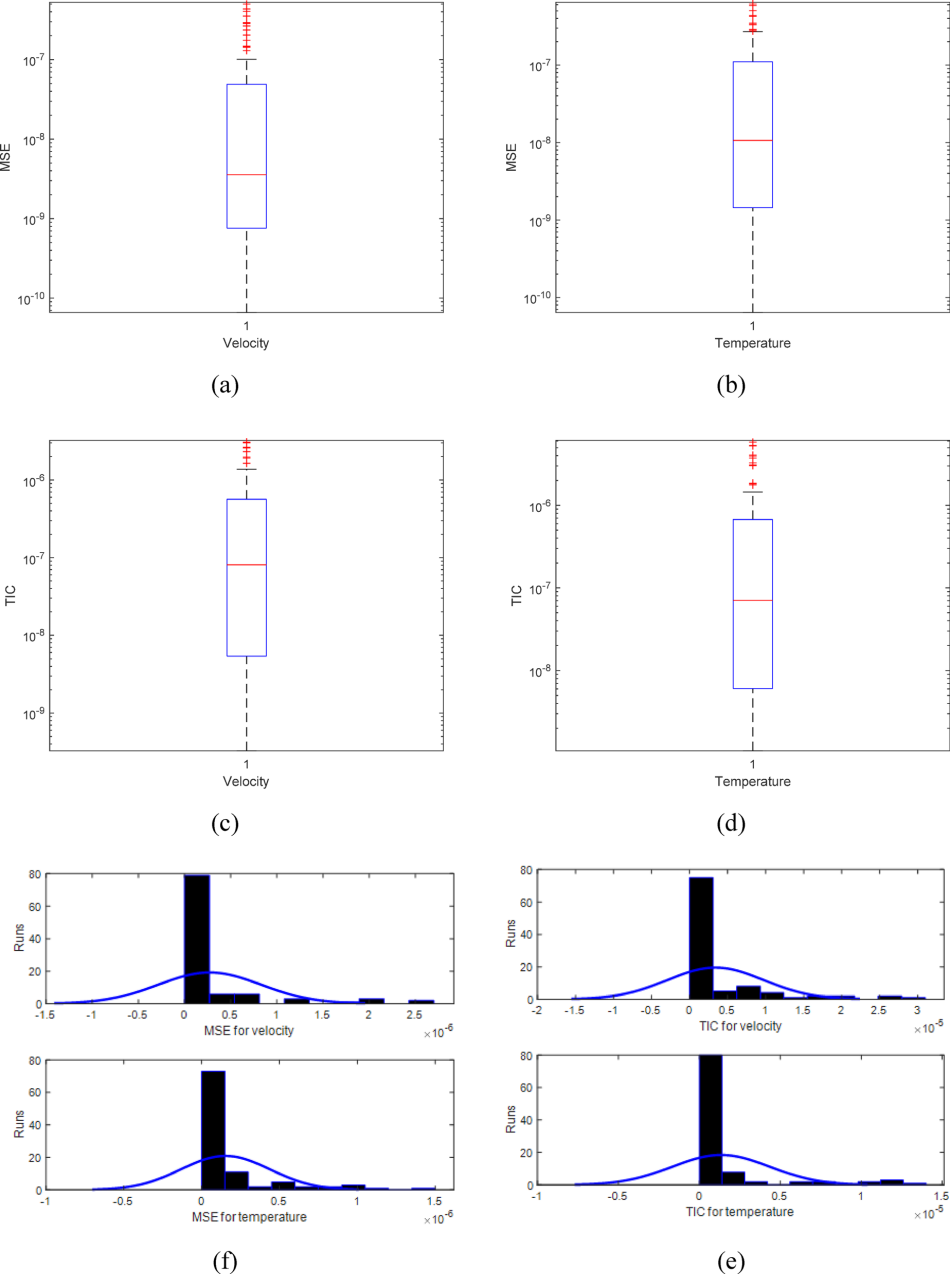
(**a**). Convergence value of MSE with 10 neurons through MTNNs for velocity profile across 100 independent runs . (**b**). Convergence value of MSE with 10 neurons through MTNNs for temperature profile across 100 independent runs (**c**). Convergence value of MSE with 10 neurons through MTNNs for velocity profile across 100 independent runs. (**d**). Convergence value of MSE with 10 neurons through MTNNs for temperature profile across 100 independent . (**e**). Convergence value of MSE with histograms for 10 neurons through MTNNs for temperature profile across 100 independent runs. (**f**). Convergence value of TIC with histograms for 10 neurons through MTNNs for temperature profile across 100 independent runs.

Furthermore, Fig. 8a,b display the rate of convergence for mean squared error (MSE) for velocity and temperature profile. The MSE values for ${u}_{f}(y)$ and ${\theta }_{f}(y)$ range from ${10}^{-07}$ to ${10}^{-09}$ and ${10}^{-06}$ to ${10}^{-09}$, respectively. Similarly, the histogram values of MSE for ${u}_{f}(y)$ and ${\theta }_{f}(y)$ are around ${10}^{-05}$ to ${10}^{-06}$ and ${10}^{-05}$ to ${10}^{-06}$, as illustrated in Fig. 8f. Figure 8c,d display the rate of convergence for Theil’s inequality coefficient (TIC) for velocity and temperature profile. The TIC values for ${u}_{f}(y)$ and ${\theta }_{f}(y)$ range from ${10}^{-05}$ to ${10}^{-08}$ and ${10}^{-05}$ to ${10}^{-08}$, respectively. Similarly, the histogram values of TIC for ${u}_{f}(y)$ and ${\theta }_{f}(y)$ are around ${10}^{-04}$ to ${10}^{-05}$ and ${10}^{-04}$ to ${10}^{-05}$, as illustrated in Fig. 8e. The Tables 2 and 3 tabulated the comparison values between the reference and proposed solutions across 100 independent runs through minimum (min), maximum (max), average and standard deviation (S.D) values for velocity and temperature.

## Conclusion

In this work, the application of artificial intelligence, particularly neural networks, to solve these equations efficiently fully explored. The MTNNs algorithm has not widely used in conjunction with neural networks for solving multi-phase wavy flow models. Moreover, the problem containing both the electroosmosis and magnetic field for pumping flow of Carreau model has been discussed. In this work, the proposed research fills these gaps by presenting an AI-based morlet wavelet artificial neural to execute multi-phase wavy flow systems, generating a more accurate and efficient solution for the considered physical problem. The major results have been detailed below:

- The proposed MTNNs hybrid approach demonstrates high accuracy, convergence, and robustness in solving the multi-phase wavy flow model, particularly with respect to the Hall-currents and EDL effects.
- The MSE values for ${u}_{f}(y)$ and ${\theta }_{f}(y)$ range from ${10}^{-07}$ to ${10}^{-09}$ and ${10}^{-06}$ to ${10}^{-09}$, respectively. Similarly, to check the convergence of proposed approach across multiple runs of histogram values of MSE for ${u}_{f}(y)$ and ${\theta }_{f}(y)$ are around ${10}^{-05}$ to ${10}^{-06}$ and ${10}^{-05}$ to ${10}^{-06}$. The TIC for ${u}_{f}(y)$ and ${\theta }_{f}(y)$ range from ${10}^{-05}$ to ${10}^{-08}$ and ${10}^{-05}$ to ${10}^{-08}$, respectively. Similarly, the histogram values of TIC for ${u}_{f}(y)$ and ${\theta }_{f}(y)$ are around ${10}^{-04}$ to ${10}^{-05}$ and ${10}^{-04}$ to ${10}^{-05}$, as for multiple runs showed the convergent.
- By integrating MTNNs with PSO the model efficiently optimizes neural network weights and biases.
- Validation against the Adam numerical reference solution, along with detailed statistical analyses, confirms the reliability of this stochastic approach in capturing the complex dynamics of the flow system.
- The presented method offers a promising tool for tackling similar multi-phase flow problems with boundary conditions and variable parameters.
- The flow velocity falls as the magnetic field parameter (M) rises.
- The flow velocity rises as the parameter (m) value falls.
- The velocity profiles show that the flow velocity falls as the parameter (k) value rises.
- It has been found that the flow velocity rises as the value of the parameter (c₁) falls.
- The fluid temperature falls as the parameter (k) value rises.
- The fluid’s temperature rises as the parameter’s (c₁) value falls.
- The fluid temperature rises when the parameter (Ue) value falls down.
- The fluid’s temperature rises as the Prandtl number (Pr) falls.
- The proposed AI technique, MTNNs, offers advantages in handling nonlinear problems due to the hybrid use of two nonlinear activation functions. However, a limitation is that it may not be effective for problems defined within the domain of quantum-based differential equations.

## Future work

In future work, we aim to solve computational fluid dynamics problems using MTNNs integrated with quantum-based optimizers. This approach will enhance solution accuracy while significantly reducing computational cost. It offers a promising direction for handling complex fluid dynamics models efficiently.

## Data Availability

The datasets used during the current study available from the corresponding author on reasonable request.

## List of symbols

$$H$$ (m/s): Walls function
$$x$$ (m): Axial coordinate
$$t$$ (S): Time coordinate
$$\lambda$$ (m): Wavelength
$${\varvec{\tau}}$$ (N/m^2^): Stress tensor
$$\varphi$$ (V): Electric potential
$${N}_{1}$$(N): Drag force
$$k$$: Electroosmostic parameter
${u}_{f},{v}_{f}$: Fluid phase velocity components
${u}_{p}, {v}_{p}$: Particulate phase velocity componets
${\theta }_{f}$: Fluid phase temperature
${\theta }_{p}$: Particulate phase temperature
${c}_{1}$: Solid particles concentration
$$Re$$: Reynolds number
$$\delta$$: Wave number
$M$: Magnetic field strength
$$m$$: Hall parameter
$Ue$: Helmholtz–Smoluchowski velocity
$${E}_{c}$$: Eckert number
$${P}_{r}$$: Prandtl number
$$\phi$$: Amplitude ratio
